# The Presence of Nucleated Red Blood Cells as an Indicator for Increased Mortality and Morbidity in Burn Patients

**DOI:** 10.1093/jbcr/irab035

**Published:** 2021-02-20

**Authors:** Phillip M Jenkins, Fadi Al Daoud, Leo Mercer, Donald Scholten, Kristoffer Wong, Vinu Perinjelil, Karl Majeske, James Cranford, Ghaith Elian, Tina Nigam, Chase A Carto, Gul R Sachwani-Daswani

**Affiliations:** 1 Hurley Medical Center, Trauma Services, Flint, Michigan, USA; 2 School of Business Administration, Oakland University, Rochester, Michigan, USA; 3 Michigan State University, College of Human Medicine, East Lansing, Michigan, USA

## Abstract

Nucleated red blood cells (NRBCs) have been studied in critically ill and injured patients as a predictor of increased in-hospital mortality and poor clinical outcomes. While prior studies have demonstrated the prognostic power of NRBCs in the critical patient, there has been a paucity of literature available describing their value as a prognostic indicator in the severely burned patient. This retrospective observational study was conducted from 2012 to 2017. Inclusion criteria for this study included all burn patients with total body surface area > 10% who were aged ≥ 15 years. Demographic and clinical data were collected from the electronic medical record system. Data analysis consisted of descriptive and comparative analysis using SPSS. Two hundred and nineteen patients (17.5%) met inclusion criteria with 51 (23.3%) patients positive for NRBCs. The presence of NRBCs had an increased mortality rate with an odds ratio of 6.0 (*P* = .001; 2.5, 14.5); was more likely to appear in older patients (*P* < .001); and was associated with increased hospital length of stay (*P* < .001), injury severity scores (*P* < .001), and complications. The presence of NRBCs even at the low concentrations reported in our study showed a 6-fold increase in the rate of mortality. With the current improvements in burn care leading to higher survival rates, the need to improve upon the numerous models that have been developed to predict mortality in severe burn patients is clear given the significantly increased risk of death that the presence of NRBCs portends.

Nucleated red blood cells (NRBCs), also known as normoblasts or erythroblasts, have been studied in critically ill and injured patients as a predictor of increased in-hospital mortality and poor clinical outcomes. The presence of NRBCs has been used as a biomarker to indicate significant hypoxic and inflammatory injuries.^1–4^ While prior studies have demonstrated the prognostic power of NRBCs in the critical patient, there has been a paucity of literature available describing the value of NRBCs as a prognostic indicator in the severely burned patient since.^5, 6^

Severe burns injuries are deep partial-thickness (second degree) and full-thickness burns involving 20% or more of the patient’s total body surface area (TBSA).^7^ These devastating injuries are among the worst forms of trauma and lead to a profound stress response, metabolic perturbations, burn-induced coagulopathy, disruption of the skin barrier, and infection.^7, 8^ NRBCs are erythropoietic progenitor cells not present in the peripheral circulation of healthy adults and children more than a month old and serve as an indicator for several diseases and disease states, such as sepsis, injuries, anemia, cancers, congestive heart failure, and many hematological disorders.^1–6, 9, 10^ Importantly, pro-inflammatory cytokines (interleukin-3 and interleukin-6) and erythropoietin, as a marker of hypoxia, are major factors in the release of NRBCs into the peripheral blood.^9, 10^ Severe burn injuries are highly inflammatory disease states for prolonged periods, up to and beyond 2 years post-injury,^8^ and depending on the presence of inhalation injuries potentially include significant hypoxia.^7, 8^

NRBCs have not been extensively evaluated as a prognostic indicator in patients with burn injuries. Severe burn injuries may represent a potentially significant interest in that both of the main releasing factors, hypoxic and inflammatory injuries, for NRBCs into the periphery are present for prolonged periods in patients with moderate to severe burn injuries. The objective of the study was to determine if the presence of NRBC in patients with ≥ 10% TBSA was associated with higher mortality and morbidity when compared with NRBC-negative patients.

## METHODS

In this retrospective observational study (2012–2017), we evaluated the prognostic value that the presence of NRBCs had on in-hospital mortality in burn patients compared with those who remained NRBC negative throughout their hospital stay. The study was conducted at our American College of Surgeons (ACS) verified Level 1 Adult and Level 2 Pediatric Trauma Center. All patients included were admitted to our burn unit.

### Data Collection

Following Institutional Review Board approval, we collected data for patients sustaining ≥ 10% TBSA from our trauma registry and patient electronic medical record (EMR) system. Inclusion criteria for this study included all burn patients with TBSA > 10% and patients aged ≥ 15 years. Data collected from the EMR included age, gender, race, TBSA, degree of burn, injury severity score (ISS), hospital length of stay (LOS), complications and comorbidity, mechanism of injury, and management. Siemens A2120I blood analyzer (BA) was used to detect the presence of NRBCs in the peripheral blood of patients. A manual verification via peripheral blood smear was conducted to confirm the presence of NRBCs flagged by the automated BA and was reported as the number of NRBCs/100 white blood cells (WBCs). NRBCs were measured from the day of admission until resolution or discharge. We also evaluated complete blood count and lactate values. 

### Statistical Analysis

Statistical analyses were conducted using SPSS (Version 27.0; IBM Corporation, Armonk, New York). For quantitative univariate variables, a two-sample *t*-test was used for comparing groups of patients on continuous variables. Fisher’s Exact test was used for comparing two groups of patients binary variables with the Chi-Squared test used for three or more groups of patients. Statistical tests were deemed significant for *P*-values less than .05.

## RESULTS

**Figure 1.**
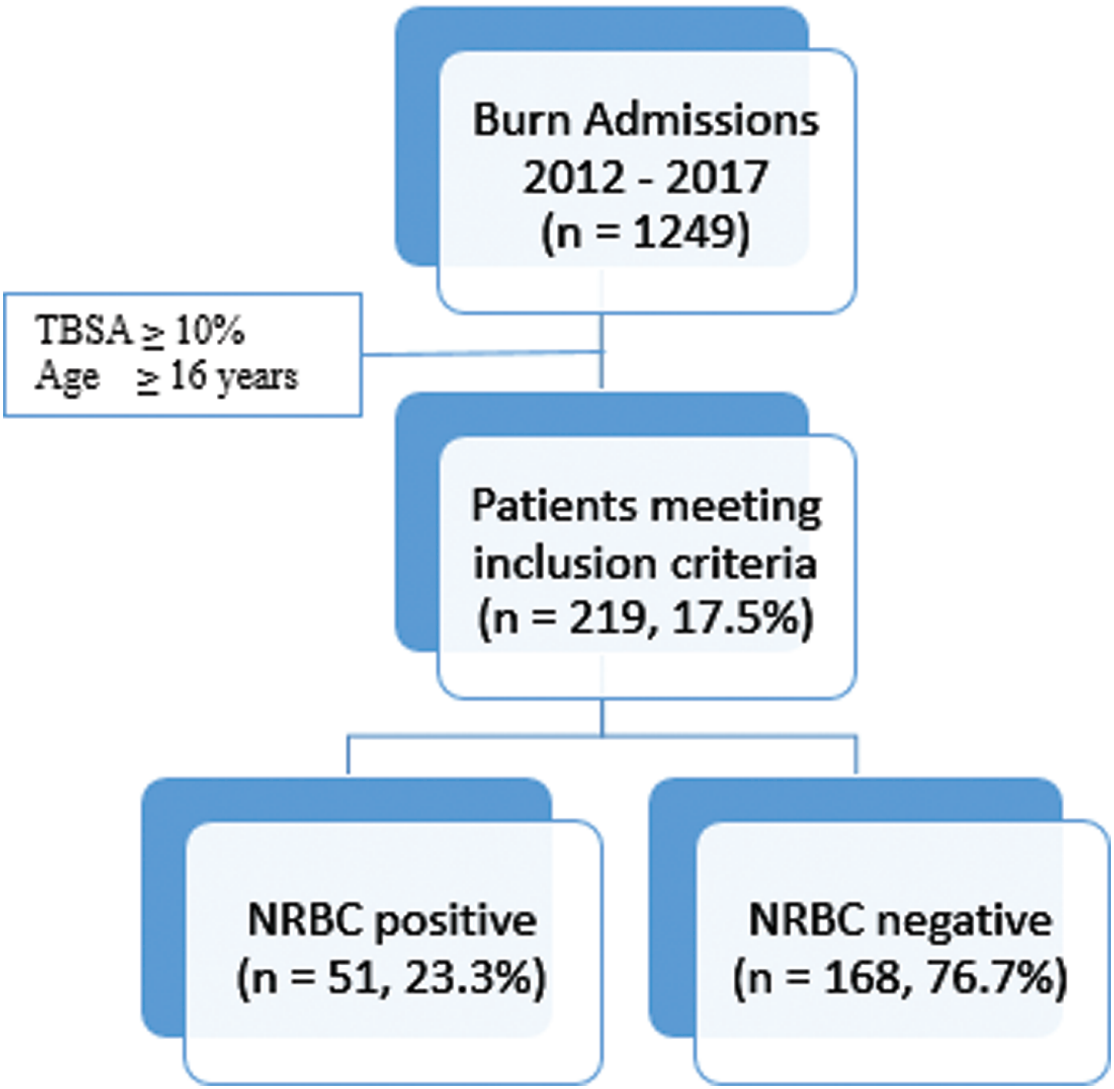
Study outline describing the total number of burn admits during the study period and those meeting inclusion criteria grouped by the presence of nucleated red blood cells (NRBCs).

A total of 1249 patients were admitted for burns from 2012 to 2017. Of those, 219 patients (17.5%) met inclusion criteria with TBSA > 10% and age ≥ 15 years, and the presence of NRBCs was detected in 51 patients (23.3%) (Figure 1). Patient demographics and clinical characteristics are presented in Table 1. The majority of patients were white (83.1%), male (79.5%), and presented with thermal injuries (90.0%). The mean ISS was 11.1 (±9.9) with an average hospital LOS of 17.3 (±22.6) days. For comorbidities, only atrial fibrillation and congestive heart failure were found to have statistical significance (*P*-value: .012 and .011, respectively). The distribution of %TBSA was as follows: 10% to 19% (n = 123; 56.2%), 20% to 30% (n = 43; 19.6%), and >30% (n = 53; 24.2%). The majority of patients sustained second- and third-degree burns (n = 205; 95%). One hundred and fifteen (52.5%) required surgical debridement and skin grafting. The remaining patients were treated with local debridement and local wound care.

**Table 1.** Patient demographics and clinical characteristics

2012–2017		
Adult burn admits		942
Study sample		219 (17.5%)
Age	mean (±SD)	45.6 (±17.9)
Gender	M	174 (79.5%)
	F	45 (20.5%)
Race/ethnicity	W	182 (83.1%)
	AA	23 (10.5%)
	Other	14 (6.4%)
Comorbidities	AFib	3 (1.4%)
	CHF	5 (2.3%)
	COPD	23 (10.5%)
	DM	21 (9.6%)
	HTN	44 (20.1%)
	Obesity	8 (3.7%)
	Smoking	96 (43.8%)
	Other	47 (21.5%)
ISS	Mean (±SD)	11.1 (±9.9)
Hospital LOS	Mean (±SD)	17.3 (±22.6)
MOI	Thermal	197 (90.0%)
	Chemical	21 (9.6%)
	Electrical	1 (0.5%)
TBSA	10–19%	123 (56.2%)
	20–30%	43 (19.6%)
	>30%	53 (24.2%)
Degree of burn	2/3	208 (95.0%)
	1/2/3	11 (5.0%)
Management	LD + WC	96 (43.8%)
	SD + Grafting	115 (52.5%)
	None	8 (3.7%)

Abbreviations: AA, African American; AFib, atrial fibrillation; CHF, chronic heart failure; COPD, chronic obstructive pulmonary disease; DM, diabetes mellitus; HTN, hypertension; ISS, injury severity score; LD, local debridement; LOS, length of stay; MOI, mechanism of injury; W, white; WC, wound care.

Bivariate analyses based on the presence of NRBCs are reported in Tables 2 and 3. Patients with positive NRBCs were older (52.9 ± 16 vs 43.7 ± 17.9; *P* = .001), had a higher ISS (18.9 ± 12 vs 8.7 ± 11.2; *P* < .001), and also had a longer hospital stay (39.8 ± 39.6 vs 10.1 ± 9.2; *P* < .001). Thermal injuries were the most common mechanism of injury noted for both groups (NRBC+ n = 50 and NRBC− n = 147). There was a larger number of NRBC+ patients with a TBSA >30% when compared with NRBC− patients, respectively (n = 32, 64.7%; n = 20, 11.9%). In both groups, there was an equivalent proportion of patients sustaining second- and third-degree burns. Patients with circulating NRBCs had a higher mortality rate when compared with patients who did not have NRBCs (n = 14/51, 27.5 % vs n = 10/168, 6%; *P* < .001). Patients who developed acute respiratory distress syndrome (ARDS; n = 10, 19.6% vs n = 3, 1.8%; *P* < .001), burn-induced anemia (mean hemoglobin value 10.1 mg/dL ± 1.6 vs 13.2 mg/dL ± 2; *P* < .001), pneumonia (n = 10, 19.6% vs n = 3, 1.8%; *P* < .001), and UTI (n = 9, 17.7% vs n = 2; 1.2%; *P* < .001) were noted to be to have a higher incidence of circulating NRBCs.

**Table 2.** Bivariate analyses evaluating the impact of nucleated red blood cells (NRBCs) in burn patients

		NRBC+	NRBC−	*P-*value
Study sample		51 (23.3%)	168 (76.7%)	
Age	mean (±SD)	52.9 (±16.0)	43.7 (±17.9)	.001
ISS	mean (±SD)	18.9 (±12.1)	8.7 (±11.2)	<.001
Hospital LOS	mean (±SD)	39.8 (±39.6)	10.1 (±9.2)	<.001
Mortality		14 (27.5%)	10 (6.0%)	<.001
MOI	Thermal	50 (98.0%)	147 (87.5%)	.039*
	Chemical	1 (2.0%)	20 (11.9%)	
	Electrical	0	1 (0.6%)	
TBSA	10–19.9%	9 (17.7%)	114 (67.9%)	<.001*
	20–29.9%	9 (17.7%)	34 (20.2%)	
	≥30%	32 (64.7%)	20 (11.9%)	
Degree of burns	2/3	48 (94.1%)	160 (95.2%)	.753*
	1/2/3	3 (5.9%)	8 (4.8%)	

Abbreviations: ISS, injury severity score; LOS, length of stay; MOI, mechanism of injury.

*Chi-square analysis.

**Table 3.** Mortality and morbidity in burn patients with nucleated red blood cells (NRBCs)

		NRBC+	NRBC−	*P*-value
Mortality		14 (27.5%)	10 (6.0%)	<.001
Complications	ACS	1 (2.0%)	2 (1.2%)	.551
	AKI	1 (2.0%)	0	.233
	ARDS	10 (19.6%)	3 (1.8%)	<.001
	CPR performed	3 (5.9%)	1 (0.6%)	.01
	C-diff	2 (3.9%)	0	.01
	DVT	3 (5.9%)	2 (1.2%)	.049
	Burn-induced anemia*	10.1 (±1.6)	13.2 (±2.0)	<.001
	MI	0	2 (1.2%)	.43
	Pneumonia	10 (19.6%)	3 (1.8%)	<.001
	PE	2 (3.9%)	0	.01
	Sepsis	7 (13.7%)	0	<.001
	UTI	9 (17.7%)	2 (1.2%)	<.001

Abbreviations: AKI, acute kidney injury; ARDS, acute respiratory distress syndrome; C-diff, clostridium difficile colitis; CPR, cardiopulmonary resuscitation; DVT, deep vein thrombus; UTI, urinary tract infections.

*Reported as mean hemoglobin (mg/dL).

For the NRBC-positive patients, we compared the NRBC profile and other laboratory values between survivors and deceased patients as presented in Table 4. The mean NRBC count for deceased patients was statistically higher than those who survived (6 ± 8.5 vs 1.4 ± 0.6; *P* = .002). Patients who died had an earlier appearance of NRBC when compared with patients who survived. While not statistically significant, the days to appearance of NRBCs were on average 5.2 (±3.4) days for deceased patients vs 9.1 (±7.9) days for survivors (*P*-value: .080) with days to resolution at 15.8 (±26.8) days for deceased patients vs 23.0 (±17.0) days for survivors (*P*-value: .255). Patients who had NRBCs that survived had a lower NRBC count (1.4 ± 0.6), and the first NRBCs were detected later in their admission and resolved by day 23. NRBC count is reported as the mean concentration over the hospital course. No statistical significance was found between the groups when considering WBC, hemoglobin, and hematocrit lab values; however, lactate with an average of 4.5 (±4.7) for deceased patients vs 1.8 (±0.5) for survivors and platelets at 171.0 (±70.6) vs 334.6 (±115.5) were significant (*P*-values: .001 and <.001, respectively).

**Table 4.** Nucleated red blood cells (NRBC) profile and other lab profiles for NRBC-positive survivors vs deceased

		Deceased	Survivor	*P*-value
NRBC positive		14 (27.5%)	37 (72.5%)	
NRBC profile				
NRBC count	Mean (±SD)	6.0 (±8.5)	1.4 (±0.6)	.002
Days to appearance	Mean (±SD)	5.2 (±3.4)	9.1 (±7.9)	.08
Days to resolution	Mean (±SD)	15.8 (±26.8)	23.0 (±17.0)	.255
Other lab profiles	WBC	11.8 (±5.8)	11.4 (±3.5)	.75
	Hemoglobin	10.6 (±2.0)	10.0 (±1.4)	.27
	HTC	32.7 (±6.0)	31.0 (±4.2)	.246
	Lactate	4.5 (±4.7)	1.8 (±0.5)	.001
	Platelets	171.0 (±70.6)	334.6 (±115.5)	<.001

Abbreviations: HTC, hematocrit; WBC, white blood cell.

## DISCUSSION

In this single-center retrospective study, we investigated the value of monitoring NRBCs as a marker for monitoring critically ill burned patients. Our study demonstrated that the presence of circulating NRBCs in severely burned patients is associated with a higher mortality rate. This is consistent with previously published studies stating that circulating NRBCs can serve as a prognostic indicator for increased mortality in critically burned patients.^2,11,12^ Despite low concentrations of NRBCs, our study revealed that the presence of NRBCs in the peripheral blood is associated with a 6-fold increased risk of death.

While the mechanism of NRBC release into the peripheral blood is not clearly understood, studies looking at the presence of NRBCs in critically ill patients suggest that hypoxia and inflammation are the key driving factors.^1–6, 9, 10, 13^ Previously, it was reported that 90% of NRBC-positive burn patients died of sepsis compared with 54% of NRBC-negative patients indicating a strong association with systemic inflammation.^6^ Also, severe hypoxemia (and inflammation) has been reported as a leading cause for the presence of NRBCs, and, even for patients with overt signs of severe disease, shock, ARDS, or severe trauma, NRBCs may be the only strong signal for disease severity.^10, 14^ While not statistically different (*P* = .08), the earlier appearance of NRBCs was linked to higher mortality and higher NRBC counts. Interestingly, we found that the following clinical characteristics (age, severity of injury, degree of burn, and TBSA), comorbidities (atrial fibrillation, chronic heart failure, and hypertension), and complications (ARDS, cardiopulmonary resuscitation performed during admission, clostridium difficile colitis, deep vein thrombus/pulmonary embolism, burn-induced anemia, pneumonia, sepsis, and urinary tract infections [UTIs]) leading to increased oxygen demand and subsequent hypoxemia had a higher rate of NRBC occurrence and the amount of NRBCs present. The anemia of critical illness especially when related to burn injuries is extremely complex. Some data suggest that erythropoiesis in the bone marrow is dampened after a burn injury, leading to a decrease in the overall erythrocyte production.^15, 16^ Given the disruption in the native mechanism of red blood cell proliferation, NRBCs (erythroblasts) begin to appear in the circulating blood. Consistent with the literature, our study demonstrates that patients with a significant reduction in hemoglobin due to the burn injury were more likely to have circulating NRBCs present when compared with patients without signs of anemia (10.1 ± 1.6 vs 13.2 ± 2, *P* < .001). Also, patients developing ARDS after sustaining burn injury had a higher incidence of circulating NRBCs (ARDS/NRBC+: 10 [19.6%] vs ARDS/NRBC−: 3(1.8%); *P* < .001). The overall incidence of ARDS patients with circulating NRBCs was 19.6%. However, from the 14 NRBC-positive patients who died, 71% developed ARDS.

As previously mentioned, severe burn injuries are highly inflammatory disease states and depending on the presence of inhalation injuries potentially include significant hypoxia. This leads to a prolonged metabolically heightened state with increased adrenergic activity, inflammatory stress, metabolic derangement, and loss of lean body mass that may be present post-injury up to and beyond 2 years. This hypermetabolic state complicates delivering the necessary energy and nutritional requirements leading to cachexia and an increase in whole body oxygen consumption compounding any hypoxic injuries that are present.^8^ The leading cause of death in burn patients is multiorgan failure and burn sepsis.^7, 8, 17, 18^ As such, NRBCs should be considered as a surrogate marker for complications following moderate to severe burns that increase the inflammatory and hypoxic burden placed on the patient. A high index of suspicion for the development of sepsis, ARDS, acute kidney injury, pneumonia, UTI, and post-burn anemia should be investigated early during the hospital course once NRBCs are present. The presence of NRBCs even at the low concentrations reported in our study showed a 6-fold increase in the rate of mortality. Furthermore, the increasing concentration of NRBCs indicates an increasing risk of mortality with several studies reporting that 2000 NRBCs/µl as an absolute indicator of mortality.^6, 11, 12, 19^ With the current improvements in burn care leading to higher survival rates, the need to improve upon the numerous models that have been developed to predict mortality in severe burn patients is clear given the significantly increased risk of death that the presence of NRBCs portends.^17, 18^

The clinical relevance of NRBCs as an indicator for in-hospital mortality is well established, but it is unclear how this information can improve poorer outcomes.^20^ NRBCs have been shown to be independent of established risk models such as acute physiology and chronic health evaluation (APACHE II) and simplified acute physiology score (SAPS II) meaning that making adjustments to these scores for the level of NRBCs led to improved prediction of outcome.^20,21^ It should be noted that the presence of NRBCs is typically delayed by several days after admission, but previous studies showed that with increasing APACHE II and SAPS II scores, there was an increase in the concentration of NRBCs present. While the early predictive ability of these scoring systems does not directly benefit from screening for the presence of NRBCs that typically appear ≥ 5 days post-injury, the presence of NRBCs combined with these scoring systems should prompt focused investigation into burn-related complications and/or intervention before those complications become clinically apparent. As stated in the literature, NRBCs may be the only indication that a complication is present. Future prospective studies to determine if accounting for NRBCs in the revised Baux score, Ryan, Smith, McGwin, Abbreviated Burn Severity Index (ABSI), Belgian Outcome of Burn Injury (BOBI), and the Fatality by Longevity, APACHE II, Measured Extent of burn, and Sex score (FLAMES) may improve the predictability of outcomes, inform interventions, and more accurately predict the point of futility in treatment.^22^

## CONCLUSIONS

Even though our study is limited by sample size and being that it is a single-center retrospective study, our study demonstrates that circulating NRBCs can be used as a potential marker for critical illness in burn patients. NRBCs can be detected as early as 5 days after admission. Early detection and identification of NRBCs in the circulating blood of patients should trigger burn clinicians to aggressively workup and treat conditions such as anemia, sepsis, and respiratory conditions (ARDS and pneumonia). Future prospective studies at our institution will seek to incorporate NRBCs into the various tools available in predicting the outcomes for severely burned patients.
